# Bridging climate and health data for decision making

**DOI:** 10.1186/s12919-025-00348-y

**Published:** 2025-10-21

**Authors:** Danil Mikhailov, Rumi Chunara, Zulma Cucunubá, Jean-Francois Lamarque, Chris Lennard, Uyi Stewart

**Affiliations:** 1 Data.Org, New York City, NY USA; 2 New York University, New York City, NY USA; 3 Pontifical Javeriana University, Bogotá, Colombia; 4 SilverLining, Washington, DC USA; 5 University of Cape Town, Cape Town, South Africa

**Keywords:** Climate change, Public health, Surveillance, Data integration, Interdisciplinary collaboration, Health equity, Epidemic intelligence, Decision support, Capacity building

## Abstract

Climate change is increasingly recognised as a public health crisis, with extreme weather events intensifying the risk of climate-sensitive diseases and placing additional strain on already vulnerable health systems. Integrating climate and health data is critical to anticipating these risks and strengthening public health preparedness and response. This report presents outcomes from the 9th session of the WHO Pandemic and Epidemic Intelligence Innovation Forum, co-hosted with Data.org, which convened experts from academia, public health, and civil society to explore barriers and solutions to integrating climate and health data for decision-making. Participants from institutions including Data.org, the University of Cape Town’s Climate System Analysis Group, New York University, Pontificia Universidad Javeriana, and SilverLining shared insights on the use of downscaled climate models, cloud-based infrastructures, and cross-sectoral collaboration.

Key themes included the need to move from a data-first to a decision-first approach; democratise access to high-resolution climate data; address inequities in funding and analytical capacity, particularly in the Global South; and foster interdisciplinary communities of practice. Challenges such as incompatible data structures, limited local capacity, and inequitable access to computational resources were addressed through innovative examples such as cloud-based climate stacks, integrated forecasting tools, and capacity-building hubs. Moving forward, the forum emphasised strengthening technical infrastructure, data interoperability, and local empowerment as essential to bridging climate and health disciplines and ensuring equitable, data-driven public health responses in a warming world.

## Background

As climate change results in more frequent and severe weather events, the occurrence of climate-sensitive diseases and climate-driven health risks is growing. It is therefore increasingly important that climate data are integrated with health surveillance data to better predict, prepare for, and respond to the increase in climate-driven health crises.

The ninth session of the WHO Pandemic and Epidemic Intelligence Innovation Forum brought together experts from a diverse array of institutions to discuss how to bridge climate and health data for public health decision-making. The session was co-hosted with Data.org [1], with the aim of highlighting the relevance of integrating climate and health data, discussing challenges faced by data practitioners, and exploring opportunities for collaboration to enhance data-driven solutions in pandemic and epidemic intelligence. Experts from Data.org, the University of Cape Town’s Climate System Analysis Group (CSAG) [2], the New York University (NYU) Center for Health Data Science [3], the Pontificia Universidad Javeriana [4] and SilverLining [5] provided insights into how climate models can be downscaled for localised decision-making. They also explored how surveillance systems can better incorporate climate data to strengthen public health resilience in the face of climate-driven health crises and the importance of improving real-time monitoring, predictive modelling, and cross-sector collaboration to enhance public health resilience and equity.

## Climate change, public health, and equity in health response

A central theme of the forum was the recognition that climate change is also a public health crisis [5]. Extreme weather events such as floods, heatwaves, and droughts are increasingly driving the spread of climate-sensitive diseases, including malaria, dengue, and cholera [6]. Climate-driven disasters also trigger population displacement, conflict, and food insecurity, further compounding the risk of disease outbreaks. These challenges are particularly severe in populations where public health systems are already overburdened, and health response infrastructure is weak [7].

Integrating climate and health data is important for enhancing surveillance systems that predict and mitigate the impacts of these risks [8]. Climate data not only contributes to our ability to anticipate disease patterns but also provides insights into population vulnerability, allowing for better allocation of resources and strengthening public health preparedness [9]. However, despite growing recognition of the intersection between climate change and public health, major challenges must be overcome to achieve effective climate and health responses [10].

Aside from technical challenges, one of the most pressing limitations is access to research funding and data accessibility. According to CSAG, despite Africa being highly vulnerable to climate change, less than 4% of global climate research funding is allocated to related topics for the continent [11, 12]. Of this limited funding, much of it is often distributed to institutions in the Global North, leaving only a minimal amount for researchers in the Global South to develop the necessary analytical tools to analyze climate-health interactions, generate region-specific insights, and inform locally relevant public health strategies. The dominance of Global North institutions in climate research also skews policy and adaptation efforts. Since most climate data is produced and analysed by institutions in the Global North, the resultant policies and adaptation strategies are often developed without fully considering local contexts and needs in the Global South.

In this context, Data.org Climateverse Consortium aims to democratise access to high-quality data by providing cloud-based computing resources, open-access data, and user-friendly analytical tools to researchers, policymakers, and institutions, particularly in the Global South. Similarly, SilverLining, a non-profit organization focused on advancing climate research and climate intervention strategies, contributes to efforts that improve access to high-resolution climate data for decision-making in the Global South. By making localized data available, SilverLining supports public health officials in assessing climate-related health risks, adapting infrastructure, and preparing communities for future challenge.

NYU’s Center for Health Data Science also exemplifies efforts to address these challenges by focusing on the connection between climate change and infectious disease outbreaks in different economic contexts. In their work with the Famine Early Warning Systems Network, they focus on linking data on extreme weather events, such as floods, with health outcomes like malnutrition and waterborne disease [13]. By integrating climate data and food insecurity indicators, the project enables health officials to forecast and pre-emptively respond to potential crises, for example, by stockpiling food and medicine before an emergency unfolds.

CSAG also plays a key role in refining global climate models to achieve higher resolution for localised projections (downscaling), helping public health officials in African countries to gain a better understanding of how climate change affects health outcomes [14]. For example, CSAG’s Heat and Health project in Johannesburg analyses the urban heat island effect and its impact on heat-related illnesses that enable informed city planning efforts, including expanding green spaces and establishing cooling centres to mitigate health risks.

## How can the health and climate sectors work better together?

Climate research should play a central role in public health preparedness and response; still, effective collaboration between the health and climate sectors requires key changes, including better data integration, infrastructure development, cross-sector collaboration, capacity building, and equity-focused approaches. A critical first step is shifting from a “data-first” to a “decision-first” approach, ensuring that climate data is tailored to meet public health needs rather than being collected and analysed in isolation. The changes needed for the two disciplines to be able to work together in terms of technical infrastructure, in terms of skills, and in terms of regulation may depend on the questions and the decisions countries need to make.

The Pontificia Universidad Javeriana is bridging the gap between the climate and health sectors by emphasising the critical role of decision-making in public health. Health officials need actionable data that directly supports specific decisions, such as how to prevent the spread of climate-sensitive diseases. CSAG also advocates for a reverse-engineering approach in climate research, where the starting point is not data, but the important climate-related questions that must be answered. By first identifying the decisions that must be made, researchers can then determine what information and data are necessary to support those decisions in a multi-stressor environment. This approach ensures that scientific data is aligned with real-world needs, leading to informed, knowledge-driven action. The real challenge lies in turning raw climate data into useful knowledge, such as climate and health vulnerability assessments and risk thresholds. This requires close links between scientists and societal actors to ensure that the data is relevant to the specific needs of those making decisions.

## Obstacles and solutions for climate and health data access, integration, and interoperability

Even when a shared objective is established, significant disparities in how climate and health data are collected, structured, and analyzed remain a key barrier to effective collaboration between the two sectors. CSAG outlined the types of data commonly used in climate research, including direct measurements from weather stations, satellites, and climate models. These data can be processed at different levels of granularity, allowing for detailed analysis such as downscaling to produce localised insights. In contrast, as the presenter for NYU pointed out, health data presents challenges as they need to account for individual exposure, which is often influenced by multiple factors, such as personal movements and behaviours. Unlike climate data, which is often automated and consistent, health data relies heavily on human input, leading to issues of privacy, data management, and organisation. Climate data benefits from open-source tools and a collaborative culture, while health data is often more restricted and dependent on proprietary software.

Another major technical challenge, as SilverLining emphasised, is the complexity and scale of Earth system models, which require enormous computational resources and generate vast data volumes—equivalent to hundreds of millions of pages for a single long-term simulation. Accessing, processing, and making sense of this immense data output is a critical hurdle, especially for regions with limited resources.

Better infrastructure and technological innovation can help address these challenges. For example, cloud computing and digital public goods offer promising solutions for overcoming these challenges. In this context, Data.org Climateverse Consortium is developing a configurable climate data stack that integrates climate and epidemiological data to forecast disease outbreaks. A climate data stack refers to a layered system of technologies, data sources, and analytical tools designed to process, integrate, and analyse climate-related information [15]. Their virtual cloud infrastructure will provide high-performance computing, allowing regions with limited resources to access the computational power needed for climate analysis. A key aspect of the stack is interoperability, enabling the integration of various data sets, including global climate data, geospatial data, and social determinants of health.

SilverLining’s Cloud for Climate initiative similarly focuses on democratising access to advanced climate models. By hosting global and localised climate models in the cloud, researchers without access to expensive supercomputers can run simulations and perform analyses. The initiative also supports the creation of new climate datasets, ensuring that researchers have context-specific information to enhance climate adaptation and health preparedness.

Additional innovative approaches were highlighted in the forum, such as NYU using remote sensing and cell phone data to better understand human experiences related to climate impacts. By tracking the movement patterns of people during extreme weather events, researchers have been able to better understand how displacement due to flooding affects the spread of diseases like cholera and malaria in West Africa. Combining real-time human mobility data with satellite climate data provides a more dynamic understanding of how populations respond to disasters and how these movements affect health outcomes. Similar methods have been used to create objective proxies for food insecurity.

## Capacity building and local empowerment

Overall, addressing climate and health challenges requires a holistic approach that integrates expertise from climate science, public health, epidemiology, and data science. A critical component of this strategy includes training local personnel in using and integrating data within decision-making frameworks.

Beyond data integration, the Climateverse Consortium focuses on capacity building, training local personnel to develop solutions that address specific community needs. The capacity-building layer ensures that local professionals are equipped with the skills and knowledge necessary to leverage climate data for practical applications in their regions. This approach is seen as part of what Data.org terms "climate justice", ensuring that communities can independently address the specific climate-related challenges they face [1]. Data.org has established three regional hubs—in the Americas and African continents and the Indian subcontinent—which have played a pivotal role in creating a globally replicable model for capacity building [16]. These hubs focus on designing systems that local communities can adopt, generating content and reusable resources that can support organisations in building long-term capacities.

Similarly, SilverLining plays a key role in building capacity in the Global South by training scientists to conduct climate research using cloud-based platforms. For example, in collaboration with the U.S. National Center for Atmospheric Research [15] and Amazon Web Services [17], they led a hands-on workshop in Rwanda with participants from all over the world, that trained researchers to run climate models on cloud-based platforms. These interactive training sessions provided foundational skills in running climate models on cloud infrastructure, enabling participants to conduct more detailed climate analyses once they had access to necessary computational resources. This initiative aligns with SilverLining’s commitment to inclusivity, ensuring that training supports scientists at different career stages and promotes gender balance in climate research. Their Global South Cloud-Based Climate Computing Hub further expands access to cloud infrastructure, training, and data tools, bridging critical knowledge and resource gaps in underserved region.

In addition to technical training, the forum highlighted the importance of fostering communities of practice that bring together researchers, policymakers, and practitioners from across disciplines. For example, Data.org has been active in creating networks of researchers in regions like India and South America, where the effects of climate change are becoming increasingly severe. NYU has further advanced this initiative by building and supporting communities of practice in East Africa. Collaborating with institutions like Moi University [18], NYU’s approach focuses on equipping local statisticians and data scientists with the skills to tackle regional data challenges, particularly those intersecting health and climate impacts. CSAG further exemplifies cross-sectoral collaboration by working directly with health professionals to translate climate data into actionable public health insights. This integration of expertise across fields ensures that climate models are used effectively to inform health interventions, demonstrating the value of interdisciplinary partnerships in improving public health resilience against climate-related threats.

These communities enable local experts to apply advanced data science techniques to address specific regional needs, creating a knowledge-sharing ecosystem that extends beyond disciplinary and geographic boundaries. Together, these efforts foster cross-sector and cross-regional collaboration, supporting a growing network of researchers who can adapt data-driven strategies to diverse local contexts, enhancing resilience in the face of climate and health challenges.

## Moving forward

In moving forward, the forum underscored the need for a sustained and strategic approach to ensure that climate and health sectors are equipped to address the challenges of a warming planet. To advance collaboration between climate and health data sectors, a key shift toward an interdisciplinary approach is essential. Decision-makers can no longer rely on isolated analyses; they require integrated insights from climate, health, and socioeconomic data to address multifaceted issues such as infectious diseases or climate-related health crises. Bridging the distinct infrastructure, skills, and data formats across these sectors requires a new model of interdisciplinarity, fostering collaboration among experts across domains. A critical element involves "multilingual" researchers, who possess expertise in both health and climate science and have comprehensive understanding of the ways climate influences health, as well as awareness of social determinants, and "data to policy translators" – professionals skilled in both policy and data science, capable of interpreting complex datasets for policymakers to streamline the decision-making process by translating technical data into actionable insights that align with policy needs.

A key challenge highlighted in the forum was the inequity in climate research, as much of this data is produced in the Global North, often at scales that do not align with localized needs in the Global South. Downscaling this data to more granular levels and democratizing access are essential steps in equipping Global South researchers to conduct analyses pertinent to their regions. To operationalize these solutions, leveraging cloud infrastructure and building data products specifically for climate and health can help researchers create accessible, context-specific insights that inform decision-making at all levels, offering more equitable access than traditional, high-cost supercomputing systems. Fostering a culture of data-sharing, standardization, and co-development of analytical frameworks will also help bridge existing gaps and improve interoperability between climate and health data systems.

Moreover, there is a pronounced need for building local capacity through targeted training. Training modules need to focus on equipping local professionals with the skills to work with data in a manner that addresses local concerns. The importance of communities of practice was also underscored to build local academic capacity through networks that foster knowledge exchange. Furthermore, strengthening partnerships between climate scientists, health experts, and policymakers will be crucial in operationalizing data-driven solutions that are both practical and contextually relevant.

As climate-driven health threats continue to intensify, prioritising interdisciplinary collaboration, local capacity-building, and technological innovation can strengthen global public health responses in an era of increasing environmental challenge.

## Data Availability

Not applicable.

## Abbreviations

WHO: World Health Organization
CSAG: Climate System Analysis Group
NYU: New York University
AWS: Amazon Web Services
NCAR: National Center for Atmospheric Research
